# Cases of Puerperal Convulsions

**Published:** 1856-02

**Authors:** J. F. Beckner

**Affiliations:** Blish Mills, Franklin Co., Missouri


ARTICLE III.—*Cases of Puerperal Convulsions.*—By J. F. Beckner, M.D., of Blish Mills, Missouri.

PROF. N. S. DAVIS, M.D., CHICAGO, ILL.:

*Dear Sir*:—Permit me to occupy some of your moments in reading a report of a case of eclampsia, treated by myself:—

Was called in the evening of Dec. 25, 1855, about ten miles from my residence, to visit Mrs. B . . ., aged 29 years, a German woman, in labor with her first child. She was rather over the medium size; squarely built; thick and medium length neck; florid countenance at present (being a stranger I do not know whether this is natural or not;) face and eyes considerably tumefied; lips much swollen; tongue protruding and bleeding; dark hair, and a very clear black eye. Ascertained that labor pains had set in about 3 o'clock, P.M., and, about one hour after, she was attacked with convulsions, which commenced with and terminated every pain, which recurred two or three times in an hour. The time intervening was occupied in raving and making efforts to escape from them. I arrived at 9 o'clock. She had just had a fit as I arrived. She was now lying in a comatose condition, pulse slow, full, and hard, breathing heavy, and accompanied with convulsive sobs. The old lady that had been officiating as accoucheur, informed me that the woman was *suonger*, and that I had better ascertain how labor was advancing. I made an examination, and found no advancement of any note. She now complained of severe headache, and was soon after thrown into most violent convulsions. As soon as I could obtain a vessel, I opened a vein, and let the blood flow freely from a large orifice, until three pints were taken. I now halted to obtain another vessel. I then took two pints more, upon which she manifested some nausea. Breathing better; pulse 125, smaller, but yet hard and irregular; skin cool, and some moisture about the face and neck; unconscious of all that occurred after I arrrived during the night. She now had another convulsion, which I thought would terminate the case. I opened the temporal artery, and took near two pints of blood, in addition to what I had taken, with no apparent advantage. I now commenced with the following formula:—

Chloroform, 1 drachm; Tinct. Musk., 3 drachms; Spts. Nitre, 3 drachms.—Ordered I drachm every fifteen minutes, and watched its effects until the chloroform showed its desirable effects.

The reason I do not use the laudanum in this formula is, I had been giving laudanum previous, with no apparent effect. The above formula was all that was given during the night. She took four doses, when she became tranquil, and slept well, to all appearances, until morning, when she aroused, and manifested some consciousness, but could not swallow anything unless it would first come in contact with the larynx. I took leave until evening, when I called again, and found her much improved. I then administered a dose of calomel and oil, and left, to call next day at 3 P.M. I called and found that the medicine operated early in the morning, and that she had been delivered of a well-formed dead infant (female.) She was doing well, to all appearance. She is now about as well as could be expected, under ordinary circumstances. I have since attended another case of puerperal convulsions, in the care of another physician. She was attacked about one hour after delivery. I gave her the chloroform and laudanum, in connection with the spts. nitre, in the following proportions:—

Chloroform, 1 drachm; laudanum, 1 drachm; spirits nitre, 4 drachms.—Ordered one-sixth of a drachm every twenty minutes, until four portions had been taken, which had the desired effect, without the use of the lance or cathartics.

His case had flooded considerable before I was called. I did not use any syaptuse in any of these cases, for the only reason—I could not obtain them. I did not use the cold applications to the head, as has been directed by all the authors that we have on this most formidable disease. After these cases were fully under the influence of the chloroform, I could not find any more use for the cold applications to the head, inasmuch as the cases above alluded to were in a moisture of perspiration, as soon as they were under a full influence of the medicine. I should be very happy to see some communications from those who have had more experience with this chloroform treatment in puerperal diseases.

*Blish Mills, Franklin Co., Mo., Jan.* 7, 1856.

